# Expression profiling of cutaneous squamous cell carcinoma with perineural invasion implicates the p53 pathway in the process

**DOI:** 10.1038/srep34081

**Published:** 2016-09-26

**Authors:** Timothy A. Warren, Natasa Broit, Jacinta L. Simmons, Carly J. Pierce, Sharad Chawla, Duncan L. J. Lambie, Gary Quagliotto, Ian S. Brown, Peter G. Parsons, Benedict J. Panizza, Glen M. Boyle

**Affiliations:** 1 Department of Otolaryngology, Head and Neck Surgery and Queensland Skull Base Unit, Princess Alexandra Hospital, Brisbane, Queensland, Australia; 2 School of Medicine, University of Queensland, Brisbane, Queensland, Australia; 3 Cancer Drug Mechanisms/Drug Discovery Groups, Department of Cell and Molecular Biology, QIMR Berghofer Medical Research Institute, Brisbane, Queensland, Australia; 4 Department of Pathology, Princess Alexandra Hospital, Brisbane, Queensland, Australia; 5 Sullivan Nicolaides Pathology, Brisbane, Queensland, Australia; 6 Envoi Pathology, Brisbane, Queensland, Australia

## Abstract

Squamous cell carcinoma (SCC) is the second most common cancer worldwide and accounts for approximately 30% of all keratinocyte cancers. The vast majority of cutaneous SCCs of the head and neck (cSCCHN) are readily curable with surgery and/or radiotherapy unless high-risk features are present. Perineural invasion (PNI) is recognized as one of these high-risk features. The molecular changes during clinical PNI in cSCCHN have not been previously investigated. In this study, we assessed the global gene expression differences between cSCCHN with or without incidental or clinical PNI. The results of the analysis showed signatures of gene expression representative of activation of p53 in tumors with PNI compared to tumors without, amongst other alterations. Immunohistochemical staining of p53 showed cSCCHN with clinical PNI to be more likely to exhibit a diffuse over-expression pattern, with no tumors showing normal p53 staining. DNA sequencing of cSCCHN samples with clinical PNI showed no difference in mutation number or position with samples without PNI, however a significant difference was observed in regulators of p53 degradation, stability and activity. Our results therefore suggest that cSCCHN with clinical PNI may be more likely to contain alterations in the p53 pathway, compared to cSCCHN without PNI.

Keratinocyte cancers are the most common form of cancer worldwide. The World Health Organization has estimated there are approximately 2–3 million cases each year, although this figure is likely underestimated^1^,^2^. The average annual increase of keratinocyte cancer incidence has been 3 to 8%^3^. Squamous cell carcinoma (SCC) is the second most common subtype and accounts for approximately 30% of all keratinocyte cancers^4^. Exposure to ultraviolet (UV) light is the strongest risk factor and in keeping with this, the sun-exposed head and neck is the most frequent site of disease^4^,^5^. The vast majority of cutaneous SCCs of the head and neck (cSCCHN) are readily curable with surgery and/or radiotherapy unless high-risk features are present^6^. Perineural invasion (PNI) is recognized as one of the high-risk features of cSCCHN, and is defined as the invasion of tumor cells into the perineural space of a peripheral nerve. In many cases, PNI is thought to precede perineural spread (PNS) where tumor cells spread along the peripheral nerve within the perineural space away from the initial point of invasion. The detection of PNI in cSCCHN denotes an aggressive tumor, and signifies a worse prognosis for the patient with higher rates of local recurrence, lymph node metastases and reduced survival^7^,^8^,^9^. Many cases of PNI are asymptomatic and involve small nerves that are only detectable by microscopy. This subtype (known as incidental PNI)^10^ is typically managed by complete surgical excision and/or postoperative radiotherapy^6^,^9^,^11^. However, PNI can progress to involve cranial nerves and ganglia and eventually to the brainstem and/or leptomeninges with poor prognosis. Once the disease manifests with clinical, radiological and/or histological evidence of spread along cranial or spinal nerves, it is termed clinical PNI. Clinical PNI is associated with a worse prognosis than incidental PNI^9^,^11^,^12^, and necessitates a more aggressive treatment approach. Overall 5-year survival for clinical PNI ranges from 56–64%^13^,^14^,^15^.

In this study, we aimed to assess expression differences between cSCCHN with or without incidental or clinical PNI. The results of our expression profiling analysis showed signatures of expression associated with activation of p53, amongst other alterations. Immunohistochemical analysis of p53 showed cSCCHN with clinical PNI to exhibit a diffuse over-expression staining pattern with no tumors showing a normal staining pattern. DNA sequencing of cSCCHN samples with clinical PNI showed potential association with p53 gain-of-function, although this was not statistically significant. Further analysis showed aberrant expression of factors known to control p53 degradation, stability and activity. Our results therefore suggest that cSCCHN with clinical PNI may be more likely to contain gain-of-function mutations in p53, or alterations in the pathway, compared to cSCCHN without PNI.

## Results

### DASL expression profiling of cSCCHN with or without PNI

We sought to define and compare the expression profiles of three patient groups, specifically those with cSCCHN, cSCCHN with incidental PNI and clinical PNI from cSCCHN. FFPE tissue blocks were assessed by a pathologist for both cSCC histology and the presence of PNI. A total of 51 cases were collected and processed for the extraction of RNA (14 cases of cSCCHN, 13 cases of cSCCHN with incidental PNI and 24 cases of clinical PNI from cSCCHN). The clinical PNI tissue specimens were from patients who underwent surgical resection of involved cranial nerve as treatment for recurrence of primary tumor in the nerve (i.e. the primary cutaneous tumor had been treated previously)^16^. In total, quality RNA was extracted in 56.89% (29/51) of FFPE samples. Twenty-four patient samples (9 samples of cSCCHN, 7 samples of incidental PNI and 8 samples of clinical PNI) were profiled (Supplementary Table S1). The presence of batch effects in the data from the sample processing, array or hybridization was assessed using Principal Component Analysis before and after normalization (Supplementary Figure S1). No batch effects were found in the dataset.

**Figure 1:**
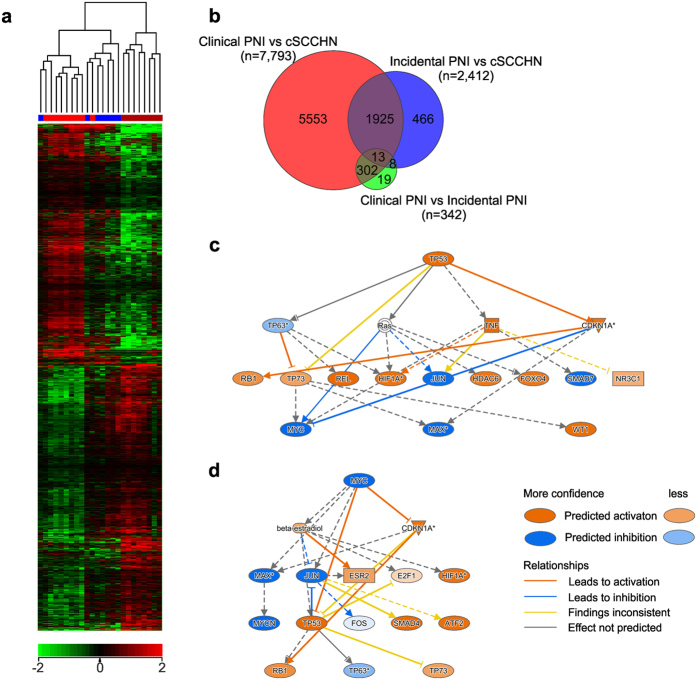
Expression profiling of cSCCHN, cSCCHN with incidental PNI or clinical PNI. (**a**) Genes found to be differentially expressed between the three groups using one-way ANOVA, *P* < 0.05, were clustered using Euclidian distance metric and Ward’s linkage, for visualisation. Red – cSCCHN, blue – cSCCHN with Incidental PNI, maroon – cSCCHN with Clinical PNI. Scale below shows difference in fold expression. (**b**) Venn diagram comparison of differentially expressed genes for pairwise analysis using t-test, *P* < 0.05 with Benjamini and Hochberg False Discovery Rate. (**c**) Mechanistic network of upstream regulator signature for differences observed in p53 signaling pathway. (**d**) Mechanistic network of upstream regulator signature for differences observed in p53 signaling pathway. Both mechanistic network figures were taken from Ingenuity Pathway Analysis software.

In total, 27,607 entities were expressed in at least one sample across the entire cohort, representing transcripts from 19,770 genes. One-way ANOVA analysis with multiple testing correction (Benjamini and Hochberg False Discovery Rate) identified 6,917 genes that showed significantly different expression between the groups (*P* ≤ 0.05). Hierarchical clustering was used to visualize these results (Fig. 1a). Genes were inversely expressed in cSCCHN without any invasion and with clinical PNI, and showed mid-level expression in disease with incidental PNI. Pairwise analyses between each pair of the three groups (t-test, *P* ≤ 0.05 with Benjamini and Hochberg False Discovery Rate) were performed. Comparison between the clinical and incidental PNI groups demonstrated 342 genes with significantly different expression (Supplementary Table S2). When clinical PNI and cSCCHN were compared, 7,793 significantly different genes were identified (Supplementary Table S3). Comparison between incidental PNI and SCC showed 2,412 genes with significantly different expression (Supplementary Table S4). There were many differentially expressed genes in cSCCHN with clinical PNI compared to cSCCHN without PNI that were not shared in the incidental PNI comparison (Fig. 1b). Although there were fewer differences between clinical PNI and incidental PNI, the majority of these differences were not found in the incidental PNI compared to cSCCHN (Fig. 1b). This suggests the possibility that clinical PNI of cSCCHN arises in a different manner or pathway to that of cSCCHN with incidental PNI.

### Pathway analysis identifies alterations in p53 signaling in cSCCHN with clinical PNI

**Figure 2:**
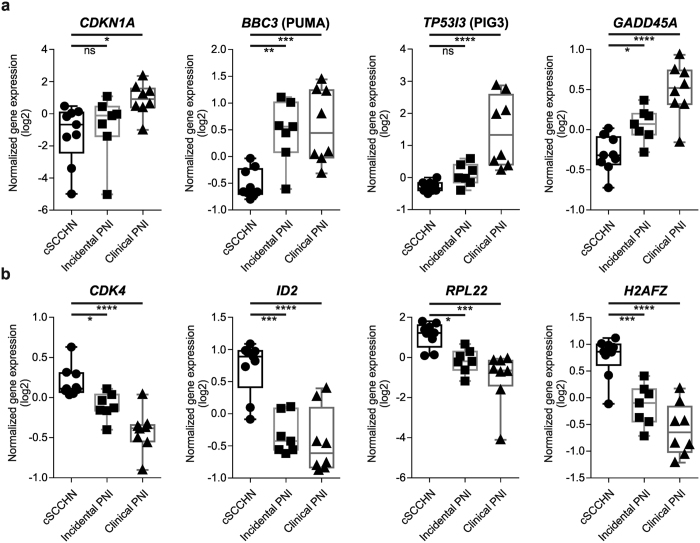
Differential expression of known p53- or MYC-target genes. Selected known target genes of (**a**) p53 or (**b**) MYC were taken from the expression profiling dataset, and plotted as box and whisker (maximum and minimum) plots. Groups are as indicated, showing normalized gene expression (log2) of each of the tumors included in the DASL analysis. Data was analyzed using one-way ANOVA with multiple comparisons. *P < 0.05; **P < 0.01; ***P < 0.001; ****P < 0.0001.

To better understand the differences in expression profile between SCC tumors exhibiting no PNI, incidental PNI and clinical PNI, we subjected the lists of genes showing differential expression to pathway analysis. We focused on upstream regulators, specifically transcriptional regulators, of the differentially expressed genes to find potential driver changes. Those with significant z-scores to infer the activation states of predicted transcriptional regulators and significant *P*-values for overlap of known targets were identified. Genes differently expressed between cSCCHN with clinical PNI and without PNI were significantly associated with apparent activation by the tumor suppressor p53, and repression of Myc when the two groups were compared (Fig. 1c,d; Supplementary Table S5). In addition, an activation associated with NOTCH1 signaling was also identified. Further, cSCCHN with incidental PNI had a signature of gene expression associated with activation of p53 and repression of Myc when compared to cSCCHN without PNI (Supplementary Table S5), although not as significantly as seen with clinical PNI. Comparison of cSCCHN with clinical versus incidental PNI identified a repression of Myc in the clinical PNI samples (Supplementary Table S5), potentially reflecting the more significant pathway repression in these tumors. The list of genes differentially expressed between cSCCHN with clinical PNI and without PNI was additionally subjected to further pathway analysis using KEGG as well as interrogation of potential promoter transcription factor binding sites using TRANSFAC and JASPAR databases, available through Enrichr^17^,^18^. Both p53 and MYC binding sites were significantly over-represented in the list of differentially expressed genes with the TRANSFAC/JASPAR analysis (Supplementary Table S6). KEGG analysis also identified a significant enrichment of genes identified in the p53 signaling pathway (Supplementary Table S7). Further analysis of the differentially expressed genes showed significantly increased expression of known p53-target genes^19^, including *CDKN1A* (p21), *BBC3* (PUMA) and *TP53I3* (PIG3) in cSCCHN with incidental and /or clinical PNI compared to tumors without PNI (Fig. 2a). There was also significantly decreased expression of well characterized MYC-regulated genes^20^, including *CDK4*, *ID2*, *RPL22* and *H2AFZ* in cSCCHN with clinical and incidental PNI compared to tumors without neural involvement (Fig. 2b).

### Immunohistochemical detection of p53 in patient tumors suggests alterations in cSCCHN with clinical PNI

**Figure 3:**
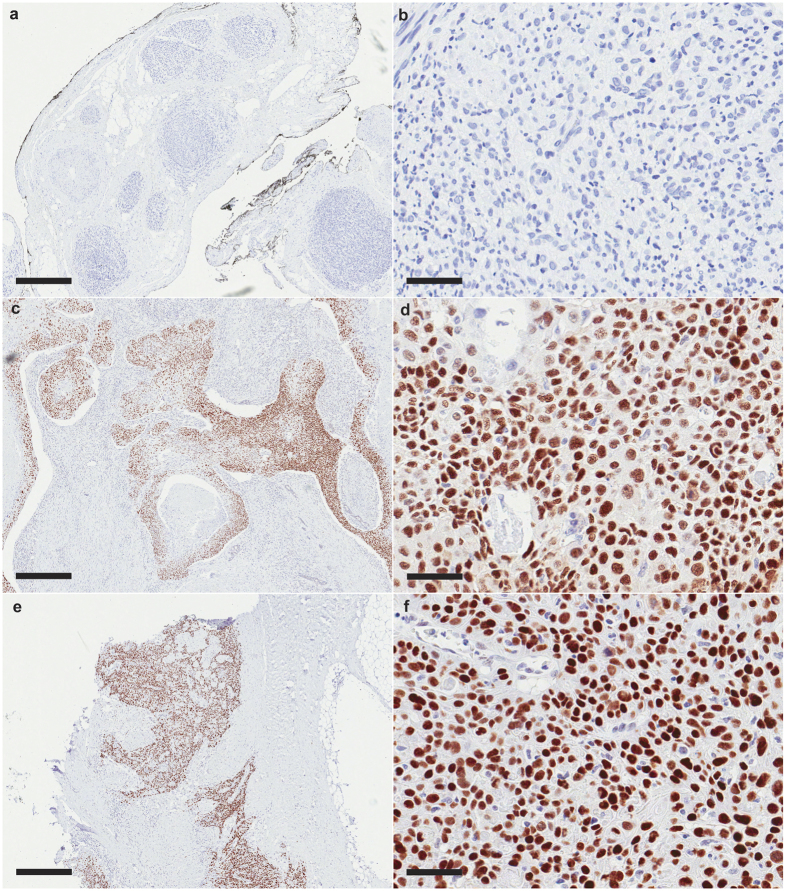
Immunohistochemical detection of p53 protein in cSCCHN with clinical PNI. Representative images of p53 staining. (**a**) A tumor with a negative pattern of p53 expression; no p53 expression is visualized in any of the tumor cells nuclei; (**b**) The same pattern shown at higher magnification; (**c**) A tumor with a focal pattern of p53 expression; strong p53 expression is evident, but within restricted areas of the tumor tissue; positively stained cells are typically immersed in a background of negative or weakly positively stained cellular nuclei; (**d**) The same pattern shown at a higher magnification; (**e**) A tumor displaying a diffuse pattern of p53 protein expression; very strong positive p53 expression scattered extensively throughout the tissue; (**f**) The same pattern shown at a higher magnification. Nuclear p53 is visualized in red. *Scale:* a, c, e: 500 μM, 40×; b, d, f: 50 μM, 400×.

To further investigate the role of p53 in PNI, we stained an independent cohort of 27 cSCCHN tumors with no PNI, 27 with incidental and 31 with clinical PNI for p53 by immunohistochemistry. Tumors were assessed for p53 staining using the criteria established by Nyiraneza and colleagues^21^ (representative images shown in Fig. 3). The analysis showed that cSCCHN with clinical PNI was significantly more likely to show a strongly positive “diffuse over-expression” pattern of p53 staining than cSCCHN without PNI (Table 1). No tumors with clinical PNI were found to show “normal” p53 staining (Table 1).

### Analysis of mutations in the p53 gene in cSCCHN with clinical PNI

Nyiraneza and colleagues^21^ identified that missense mutations were prevalent in sections with diffuse staining for p53. To determine if the same pattern could be identified within our samples, direct sequencing of *TP53* exons 4 to 8 of DNA extracted from FFPE sections of selected samples was performed. We found *TP53* mutations in 5 of 17 cSCCHN, compared to 7 of 21 cSCCHN with clinical PNI. Although the mutation number was not significantly different, it was of interest to note that some of the *TP53* mutations observed in the cSCCHN samples with clinical PNI were either at (p.G245), or directly adjacent to (p.P152; p.H193), positions previous identified as p53 gain-of-function mutations (p.P151; p.L194)^22^,^23^ (Supplementary Table S8). However, the differences in number of potential gain-of-function mutations were again not significant.

### Differential expression of regulators of p53 degradation, stability and activity in cSCCHN with clinical PNI

**Figure 4:**
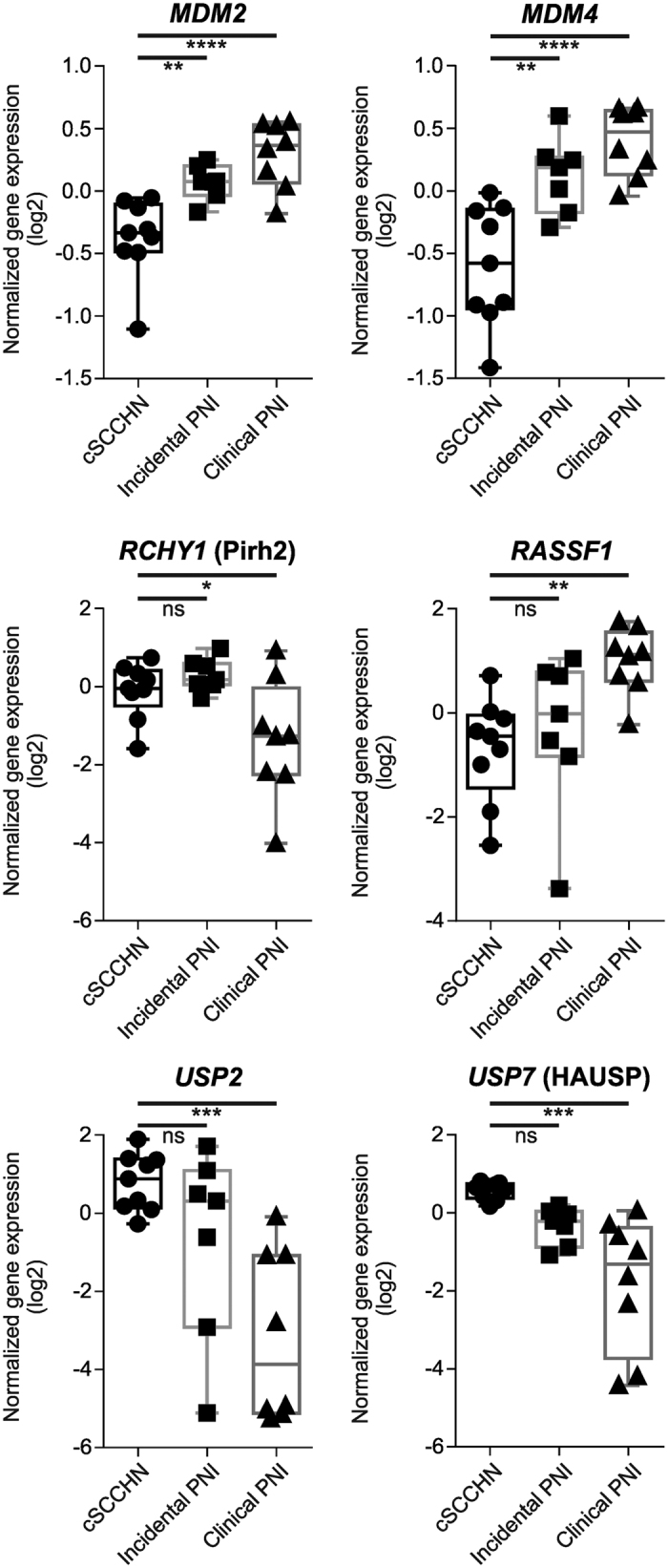
Differential expression of regulators of p53 degradation, stability and activity in cSCCHN with clinical PNI. Selected target genes known to play roles in degradation, stability and activity of p53 were taken from the expression profiling dataset, and plotted as box and whisker (maximum and minimum) plots. Groups are as indicated, showing normalized gene expression (log2) of each of the tumors included in the analysis. Data was analyzed using one-way ANOVA with multiple comparisons. *P < 0.05; **P < 0.01; ***P < 0.001; ****P < 0.0001.

To further investigate the differences in p53 staining in cSCCHN with clinical PNI in the absence of significant differences in mutational burden or position, we examined the expression of known regulators of p53 degradation and stability. We observed significant increases in expression of *MDM2*, a molecule known to target p53 for ubiquitination and degradation^24^, in cSCCHN with either incidental or clinical PNI as well as in expression of *MDM4*, a related inhibitor of p53 activity^25^ (Fig. 4). In contrast, another E3 ubiquitin ligase *RCHY1* (Pirh2) was significantly down-regulated in cSCCHN with clinical PNI. Most interestingly, the deubiquitinating enzymes encoded by *USP2* and *USP7* (HAUSP) that target MDM2 were significantly down-regulated in tumors with clinical PNI (Fig. 4). Expression of *RASSF1*, the product of which disrupts the interaction between MDM2 and HAUSP was significantly up-regulated in the clinical PNI samples. These results suggest the balance of molecules controlling p53 degradation, stability and activity is altered in cSCCHN with clinical PNI.

## Discussion

In this study, we investigated changes in gene expression in clinical and incidental PNI of cSCCHN. A spectrum of disease has previously been thought likely to exist, where cSCCHN tumors progress to cSCCHN with incidental PNI, which finally progress to tumors exhibiting clinical PNI. However, there remains an important prognostic distinction between incidental and clinical PNI of cSCCHN. Significant differences in local control and disease specific survival following treatment have been reported^26^. Our expression profiling results may not support a progression of disease. Although tumors with incidental or clinical PNI showed the least number of significant differences, the majority were not shared in the comparisons to cSCCHN without PNI (Fig. 1b). The differences in location from which our samples were taken is a limitation of our study. The samples of cSCCHN with clinical PNI were collected from the large cranial nerves (the definition of perineural spread), while the cSCCHN samples and those with incidental PNI were collected from the primary tumor site. However, in each case the primary tumor site of those patients with clinical PNI was previously removed by surgery, leaving limited alternatives. The difference in location the samples were taken from remains a potential confounding factor. In addition, our recent work has identified a tendency for cSCCHN with clinical PNI to be moderately or poorly differentiated, when compared to cSCCHN without neural involvement^16^.

Immunohistochemical detection of p53 showed cSCCHN with clinical PNI were more likely to exhibit a diffuse over-expression staining pattern, based on the previous published criteria^21^. In contrast, there was no significant difference in proportions of mutations predicted by p53 staining between cSCCHN without PNI and incidental PNI. There were no cSCCHN with clinical PNI that showed a “normal” p53 staining pattern. However, some data does support a spectrum of disease, in contrast with the above results. Pathway analysis showed a signature that was significantly associated with an activation of p53, although the significance was somewhat reduced for tumors with incidental PNI. Five of seven tumors with clinical PNI had *TP53* mutations that were either at, or directly adjacent to, positions previously implicated in p53 gain-of-function^22^. However, these findings were not significantly different to those observed in cSCCHN without neural involvement. Recent studies have suggested *TP53* to be mutated in approximately 30–70% of cSCC^27^,^28^. In contrast, aggressive cSCC tumors, including tumors with perineural invasion, were found to have a higher frequency of *TP53* mutations (94.8%)^29^. Although no mutations were found in a high proportion of cSCCHN in our study, it is important to note that only exons 4 to 8 of *TP53* were sequenced in this study, using a relatively insensitive method. Interestingly, the study comparing aggressive and non-aggressive cSCC also found that cSCC tumors with PNI were significantly associated with mutations in *NOTCH2*, and there was a non-significant trend for these tumors to also be mutated for *TP53* and *NOTCH1*^29^. However, our study has identified signatures mimicking activation of both p53 and NOTCH1. Our results highlight the need to further investigate the p53/NOTCH pathway axis in aggressive cSCC, particularly with PNI.

Additional analysis of the expression profiling data found altered levels of molecules known to regulate the degradation, stability and activity of p53. We found significant increases in expression of *MDM2* and *MDM4*, which target p53 for ubiquitination and degradation or inhibit its activity respectively. Higher levels of MDM2 and MDM4 would presumably result in less p53 and therefore reduced pathway activity. However, in contrast we found significantly reduced levels of the deubiquitinating enzymes encoded by *USP2* and *USP7* in cSCCHN with clinical PNI compared to tumors with incidental PNI or no neural involvement. Both USP2 and USP7/HAUSP target MDM2 and lead to its degradation^25^. It is also interesting to note that USP7 also protects p53 from degradation^30^,^31^. Expression of *RASSF1* was also significantly up-regulated in the clinical PNI samples. The RASSF1 protein disrupts the interaction between MDM2, DAXX and USP7/HAUSP^32^, which would promote MDM2 ubiquitination and therefore result in stabilization of p53 and increased activity. These results presented here may suggest the complex balance of molecules controlling degradation, stability and activity of p53 is disrupted in cSCCHN with clinical PNI.

We speculatively propose that tumors with clinical PNI are more likely to contain either potential p53 gain-of-function mutations, or other changes of p53 regulation leading to apparent pathway activation. Although our analysis may not support the step-wise progression of disease through tumors with incidental neural involvement, this possibility cannot be ruled out at this time. Overall, our findings may suggest an alternative pathway could be responsible for tumors exhibiting clinical PNI when compared to those with incidental PNI. Our future work will further characterize molecules involved in regulation of p53 activity to better understand the role of the p53 pathway in the process of perineural invasion.

## Methods

### Tissue specimens

This study involved patients with cSCCHN treated between 2003 and 2011. Informed consent was obtained from all participants. The study protocol was approved by the Metro South Human Research Ethics Committee (HREC Approval Number 2003/197). Specimens from patients stored as formalin-fixed paraffin-embedded (FFPE) tissue blocks were retrieved from the Princess Alexandra Hospital Department of Pathology (Brisbane) and Sullivan Nicolaides Pathology (Brisbane). All methods were carried out in accordance with the approved guidelines.

### RNA extraction from FFPE sections and quantitative real-time PCR pre-qualification analysis

Six sections of 5 μm thickness were cut from FFPE blocks and placed on superfrost slides (QIMR Berghofer Histology Unit, Brisbane). One slide was stained with haematoxylin and eosin using standard protocol. The presence and location of cSCCHN and PNI was confirmed and marked on this section by a pathologist (I.B.). Using the H&E slide as reference, tumor was macro-dissected from each slide and pooled. Total RNA was extracted and purified including an RNase-free DNase I digestion step using the RNeasy^®^ FFPE Kit (Qiagen, Hilden, Germany), and quantified (NanoDrop^®^ ND-1000; Thermo Scientific, Waltham, USA). RNA from each sample was then divided into two aliquots of 200 ng RNA, one aliquot was used in pre-qualification analysis, and the other reserved for expression profiling.

Pre-qualification and quantification of RNA was performed with real time-PCR (RT-PCR) using primer sets designed to amplify a fragment of *RPL13A*, a highly expressed ribosomal protein (F: 5′-GTACGCTGTGAAGGCATCAA-3′; R: 5′-GTTGGTGTTCATCCGCTTG-3′). Reverse transcription was undertaken to generate cDNA from RNA using a DASL Single Use cDNA Synthesis Kit (Illumina^®^, San Diego, USA) as per the manufacturer’s instructions. Each cDNA product was amplified using the Qiagen QuantiTect^®^ SYBR^®^ Green PCR Kit and a RotorGene 6000 real-time cycler (Corbett Life Science, Australia). PCR reaction conditions were: 95 °C for 15 min, followed by 40 cycles of 95 °C for 30 sec, 60 °C for 30 sec and 72 °C for 30 sec. The cycle threshold (C_T_) was calculated, and used as an estimate of target cDNA abundance with a C_T_ value ≤28 accepted as sufficient.

### Expression profiling and analysis

Whole genome expression profiling was undertaken using the Illumina^®^ Whole-Genome DASL^®^ HT Assay as per the manufacturer’s instructions. Briefly, 200 ng of total RNA per sample was reverse transcribed to cDNA using biotinylated primers with a DASL^®^ Single Use cDNA Synthesis Kit (Illumina). The biotinylated cDNAs were annealed to two DASL^®^ Assay Pool (DAP) oligonucleotides (one upstream-specific and one downstream-specific) and combined with hybridization reagent. This mixture was bound to streptavidin-conjugated paramagnetic particles to capture cDNA-oligo complexes. Following hybridization, unbound oligos were removed by washing. The hybridized cDNA was extended and ligated to create a PCR template. This template was used in a PCR reaction with two universal primers (one biotinylated, one fluorescent) to create a labeled product, which was then hybridized to the Illumina^®^ HumanHT-12 v4.0 Expression BeadChip overnight. The BeadChip was then washed and scanned using an iScan Reader (Illumina). Data was extracted using GenomeStudio (Illumina) and analyzed in GeneSpring (v12.5, Agilent Technologies, Santa Clara, USA) software. Expression values were normalized using quantile normalization with default settings. The absence of batch effects from the sample processing, array or hybridization was assessed using Principal Component Analysis before and after normalization (Supplementary Figure S1). One-way analysis of variance (ANOVA) was applied to identify differential gene expression between all study groups; pairwise t-tests were also used to compare between two groups, in each case using the Benjamini and Hochberg False Discovery Rate for multiple testing correction. Significant pathways within the data were assessed using Ingenuity Pathway Analysis (IPA^®^, Qiagen) software.

### Immunohistochemistry

Tissue sections were de-waxed, rehydrated and incubated in 2% hydrogen peroxide for 10 min. Antigen retrieval was performed in 10 mM citrate buffer for 8 min at 121 °C. Sections were cooled, washed in TBS, and blocked with Background Sniper (Biocare Medical, Concord, USA) for 15 min at RT. The p53 primary antibody (clone CM5p, Novacastra, Nussloch, Germany) was then applied overnight at RT, and MACH1 Universal Polymer (Biocare Medical) applied for 45 min following. Sections were counterstained in haematoxylin, washed in water, dehydrated, cleared with xylene and mounted. Staining of p53 was assessed according to previously published studies^21^ by a histopathologist (I.B.) blinded to the study hypothesis. Chi-squared test was used to assess differences in groups, data was considered statistically significant when p ≤ 0.05.

### DNA extraction from FFPE sections, and p53 mutation detection by DNA sequencing

Five sections of 10 μm thickness were cut from each FFPE block, and tumor macro-dissected as described above. DNA was extracted using the QIAamp^®^ DNA FFPE Tissue Kit (Qiagen). Samples were quantified using a spectrophotometer (NanoDrop^®^ ND-1000) before polymerase chains reactions (PCR) performed to amplify exons 4–8 of the *TP53* gene. PCR primers and conditions utilized were as previously described^33^. PCR products were resolved by electrophoresis, purified using the QIAquick^®^ PCR purification Kit (Qiagen) and quantified using a spectrophotometer. Products were sequenced using the BigDye^®^ v3.1 Sequencing Kit (Thermo Scientific).

## Additional Information

**Accession code:** The data discussed in this publication have been deposited in NCBI’s Gene Expression Omnibus, and are accessible through GEO Series accession number GSE86544.

**How to cite this article**: Warren, T. A. *et al*. Expression profiling of cutaneous squamous cell carcinoma with perineural invasion implicates the p53 pathway in the process. *Sci. Rep.* **6**, 34081; doi: 10.1038/srep34081 (2016).

## Supplementary Material

Supplementary Information

Supplementary Table S2

Supplementary Table S3

Supplementary Table S4

Supplementary Table S5

Supplementary Table S6

Supplementary Table S7

## Figures and Tables

**Table 1:** Immunohistochemical detection of p53 in patient tumors suggests alterations in cSCCHN with clinical PNI.

Staining Pattern	cSCCHN (%)	cSCCHN Incidental PNI (%)	*P*	cSCCHN Clinical PNI (%)	*P*
	n = 27	n = 27		n = 31	
Normal	4 (14.8%)	5 (18.5%)	0.2599	0 (0%)	<0.0001
Diffuse Overexpression	11 (40.7%)	9 (33.3%)		18 (58.1%)	
Focal/Restricted Overexpression	9 (33.3%)	9 (33.3%)		6 (19.4%)	
Negative	3 (11.1%)	4 (14.8%)		7 (22.6%)	

Staining of p53 was assessed according to previously published studies^21^ by a histopathologist (I.B.) blinded to the study hypothesis. Chi-squared test was used to assess differences in groups, data was considered statistically significant when *P* ≤ 0.05.
