# Supplementary material for: Expression profiling of cutaneous squamous cell carcinoma with perineural invasion implicates the p53 pathway in the process
Source: Sci Rep. 2016 Sep 26;6:34081. doi: 10.1038/srep34081 (PMC5035993; doi:10.1038/srep34081)
Supplement: Supplementary Information [file srep34081-s1.pdf]

Expression profiling of cutaneous squamous cell carcinoma with perineural invasion implicates the p53 pathway in the process.

Timothy A. Warren[1,2,3,7], Natasa Broit[3,7], Jacinta L. Simmons[3], Carly J. Pierce[3], Sharad Chawla[1,2,3], Duncan L.J. Lambie[4], Gary Quagliotto[5], Ian S. Brown[6], Peter G. Parsons[3], Benedict J. Panizza[1,2,7]*, Glen M. Boyle[3,7]*

[1] Department of Otolaryngology, Head and Neck Surgery and Queensland Skull Base Unit, Princess Alexandra Hospital, Brisbane, Queensland, Australia

[2] School of Medicine, University of Queensland, Brisbane, Queensland, Australia

[3] Cancer Drug Mechanisms / Drug Discovery Groups, Department of Cell and Molecular Biology, QIMR Berghofer Medical Research Institute, Brisbane, Queensland, Australia

[4] Department of Pathology, Princess Alexandra Hospital, Brisbane, Queensland, Australia

[5] Sullivan Nicolaides Pathology, Brisbane, Queensland, Australia

[6] Envoi Pathology, Brisbane, Queensland, Australia

[7] Equal contribution

*** Corresponding authors** - Benedict J. Panizza, Department of Otolaryngology, Head and Neck Surgery & Queensland Skull Base Unit, Princess Alexandra Hospital, 199 Ipswich Road, Woolloongabba, Brisbane, Queensland, Australia, 4102. Tel.: +61 7 3839 2967; Fax: +61 7 3839 0070; E-mail: bpanizza@bigpond.net.au; Glen M. Boyle, QIMR Berghofer Medical Research Institute, Locked Bag 2000, Post Office Royal Brisbane Hospital, QLD 4029, Australia. Tel.: +61 7 3362 0319; Fax: +61 7 3845 3508; E-mail: Glen.Boyle@qimrberghofer.edu.au

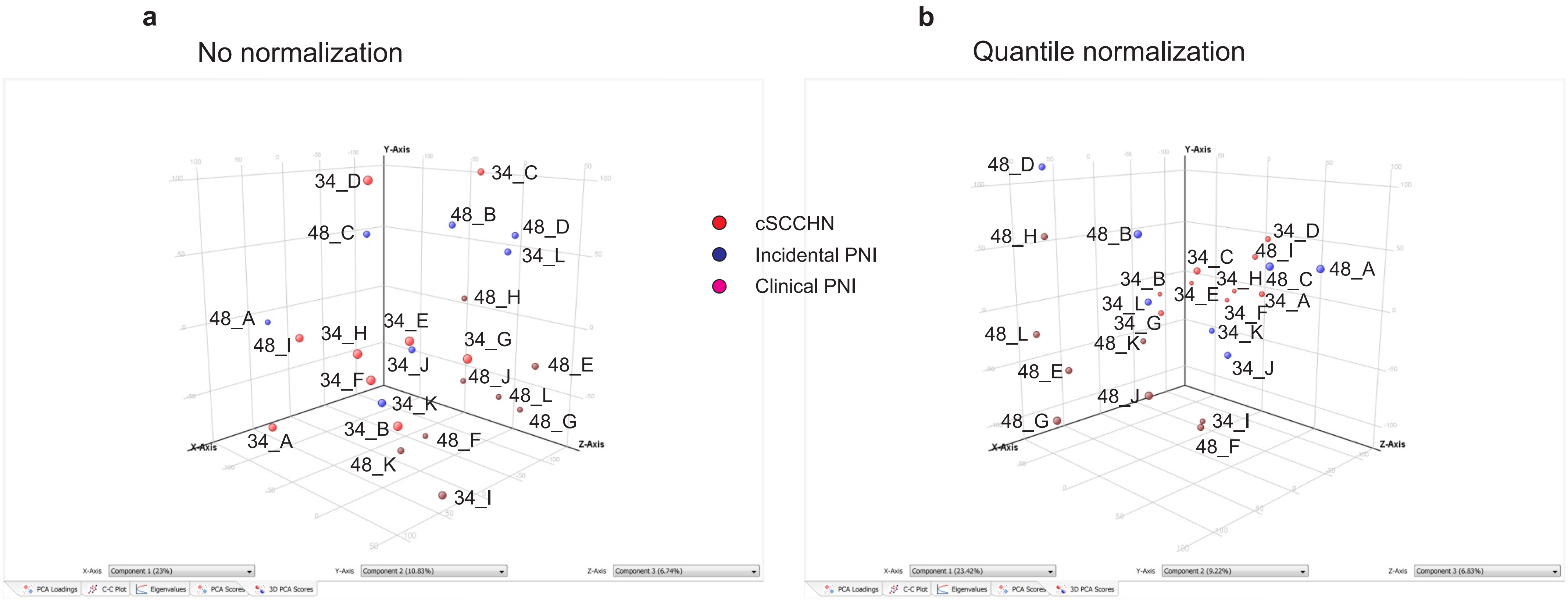

Supplementary Figure S1. Principal Component Analysis (PCA) of DASL expression profiling data before and after normalization. (**a**) PCA on samples before normalization. (**b**) PCA on samples after quantile normalization. The last characters of the chip numbers, corresponding to samples numbers shown in Table S1 are shown. Red spheres - cSCCHN without neural invasion, blue spheres - cSCCHN with Incidental PNI, maroon spheres - cSCCHN with Clinical PNI.

Array ID	Histology	Age	Gender	Collection	Grade	Primary Site	PNI	PNI site
6508593034_A	SCC	68	M	08/12/2010	Mod diff	Ear	no	n/a
6508593034_B	SCC	78	M	21/12/2010	Mod diff	Forehead	no	n/a
6508593034_C	SCC	87	M	08/12/2010	Mod-well diff	Ear	no	n/a
6508593034_D	SCC	66	M	22/12/2010	Mod diff	Cheek	no	n/a
6508593034_E	SCC	76	M	14/12/2010	Mod diff	Cheek/Zygoma	no	n/a
6508593034_F	SCC	82	F	20/12/2010	Mod diff	Neck	no	n/a
6508593034_G	SCC	71	M	22/11/2010	Mod diff	Scalp	no	n/a
6508593034_H	SCC	79	M	08/07/2011	Mod-well diff	Temple	no	n/a
6508593048_I	SCC	87	M	01/02/2011	Mod diff	Scalp	no	n/a
6508593034_J	SCC incidental PNI	71	M	08/02/2010	Mod diff	Neck	micro (<0.1mm)	local
6508593034_K	SCC incidental PNI	53	M	10/11/2010	Mod diff	Lower eyelid	micro (0.1mm)	local
6508593034_L	SCC incidental PNI	70	M	12/05/2010	Mod diff	Temple	micro (0.2mm)	local
6508593048_A	SCC incidental PNI	68	F	25/11/2010	Well diff	Nose	micro (0.1mm)	local
6508593048_B	SCC incidental PNI	79	M	19/05/2010	Poorly diff	Cheek	micro (0.1mm)	local
6508593048_C	SCC incidental PNI	77	M	06/10/2010	Mod diff	Cheek	micro (0.1mm)	local
6508593048_D	SCC incidental PNI	70	M	15/03/2010	Mod-poorly diff	Neck	micro (<0.15mm)	local
6508593034_I	SCC clinical PNI	72	M	02/11/2010	Mod-well diff	Parotid skin	clinical (0.3mm)	Parotid gland: VII
6508593048_E	SCC clinical PNI	63	M	04/09/2007	Mod diff	Unknown*	clinical (?diam)	Parotid gland: VII
6508593048_F	SCC clinical PNI	52	M	20/06/2009	Well diff	Unknown*	clinical (0.5mm)	V3 & VII
6508593048_G	SCC clinical PNI	62	M	10/04/2006	Poorly diff	Cheek/Temple	clinical (0.6mm)	Parotid gland: VII
6508593048_H	SCC clinical PNI	73	M	03/12/2010	Mod diff	Unknown*	clinical (?diam)	ITF: Trigeminal V3
6508593048_J	SCC clinical PNI	55	M	17/07/2009	Poorly diff	Preauricular	clinical (0.25mm)	Parotid gland: VII
6508593048_K	SCC clinical PNI	71	F	29/09/2009	Mod diff	Unknown*	clinical (?diam)	SPF/PPF: V2
6508593048_L	SCC clinical PNI	77	M	18/09/2009	Poorly diff	Cheek	clinical (2mm)	PPF: V2

Supplementary Table S1: Details of Patient Tumors profiled by DASL.
*In cases of clinical PNI, the likely primary site of the tumor is often treated months to years before symptoms of PNI become evident. A previous study has indicated that 12% of PNI cases have no known current or historically identifiable primary (15). Mod, moderately; diff, differentiated.

Supplementary Table S2: Differential Gene Expression between Clinical PNI versus Incidental PNI Samples. Differential expression was determined using pairwise t-tests with the Benjamini and Hochberg False Discovery Rate for multiple testing correction. Please see Excel spreadsheet.

Supplementary Table S3: Differential Gene Expression between Clinical PNI versus cSCCHN Samples. Differential expression was determined using pairwise t-tests with the Benjamini and Hochberg False Discovery Rate for multiple testing correction. Please see Excel spreadsheet.

Supplementary Table S4: Differential Gene Expression between Incidental PNI versus cSCCHN Samples. Differential expression was determined using pairwise t-tests with the Benjamini and Hochberg False Discovery Rate for multiple testing correction. Please see Excel spreadsheet.

Supplementary Table S5: Upstream Regulator Analysis of Differentially Expressed Genes from Pairwise Comparisons. Each gene list shown in Supplementary Tables S2-4 were analysed using the Upstream Regulator prediction in Ingenuity Pathway Analysis software. Each list is shown in a different spreadsheet. Please see Excel spreadsheet.

Supplementary Table S6: TRANSFAC and JASPAR Analysis of Differentially Expressed Genes from Clinical PNI versus cSCCHN Pairwise Comparison. Predicted transcription factors leading to the observed signature were predicted using TRANSFAC and JASPAR software. Please see Excel spreadsheet.

Supplementary Table S7: KEGG Analysis of Differentially Expressed Genes from Clinical PNI versus cSCCHN Pairwise Comparison. Predicted pathway alterations in the observed signature were predicted with KEGG database. Please see Excel spreadsheet.

cSCCHN										
**#**	**Exon mutated**	**ChromChange**	**Mutation Type**	**Codon Change**	**AA Change**	**Protein**	**Known?**	**#**	**IHC Stain**	**COSMIC Comments**
**1**	None								Focal	
**2**	None								Diffuse	
**3**	**Exon 6**	c.580C>T	SUB	CTT > TTT	L > F	p.L194F	Yes	24	Diffuse	Substitution - Missense
	**Exon 8**	c.797G>A	SUB	GGA > GAA	G > E	p.G266E	Yes	69		Substitution - Missense
	**Exon 8**	c.868C>T	SUB	CGC > TGC	R > C	p.R290C	Yes	8		Substitution - Missense
**4**	**Exon 5b**	c.531_532delCCInsTG	INDEL	CCC > CCT	None	p.P177P	No	0	Diffuse	Unknown
	**Exon 5b**			CAC > GAC	H > D	p.H178D				
	**Exon 8**	c.843_844delCCInsTT	INDEL	GAC > GAT	None	p. D281D	Yes	1		Substitution - Missense
	**Exon 8**			CGG > TGG	R > W	p.R282W				
**6**	**Exon 8**	c.856G>A	SUB	GAA > AAA	E > K	p.E286K	Yes	89	Focal	Substitution - Missense
**7**	None								Focal	
**8**	None								Diffuse	
**9**	None								Diffuse	
**10**	**Exon 6**	c.590T>G	SUB	GTG > GGG	V > G	p.V197G	Yes	15	Focal	Substitution - Missense
**39**									Normal	
**40**									Normal	
**41**	**Exon 6**	c.585_586delCCInsTT	INDEL	ATC>ATT	None	p.I195I	Yes	7	Focal	Substitution - Nonsense
				CGA > TGA	R > stop	p.R196end				
	**Exon 5b**	c.534_535delCCInsTT	INDEL	CAC > CAT	None	p.H178H	Yes	9		Substitution - Missense
				CAT > TAT	H > Y	p.H179Y				
**42**	None								Diffuse	
**43**	None								Focal	
**44**	None								Negative	
**45**	None								Diffuse	
**46**	None								Negative	

Supplementary Table S8. TP53 mutation detection in patient samples with cSCCHN without neural involvement or cSCCHN with clinical PNI.

Clinical PNI										
**#**	**Exon mutated**	**Location**	**Mutation Type**	**Codon Change**	**AA Change**	**AA Change**	**Known?**	**#**	**IHC Stain**	**COSMIC Comments**
**12**	**Exon 5a**	c.454C>T	SUB	CCG >TCG	P > S	p.P152S	Yes	26	Focal	Substitution - Missense
**13**	**Exon 6**	c.577C>G	SUB	CAT > GAT	H > D	p.H193D	Yes	11	Negative	Substitution - Missense
**14**	None								Diffuse	
**15**	None								Negative	
**16**	None								Diffuse	
**17**	None								Focal	
**18**	**Exon 6**	c.577C>G	SUB	CAT > GAT	H > D	p.H193D	Yes	11	Diffuse	Substitution - Missense
	**Exon 7**	c.733_734delGGInsAA	INDEL	GGC > AAC	G > N	p.G245N	Yes	2		Substitution - Missense
**19**	None								Diffuse	
**20**	None								Diffuse	
**26**	None								Diffuse	
**28**	**Exon 8**	c.899C>T	SUB	CCC > CTC	P > L	p.P300L	Yes	6	Diffuse	Substitution - Missense
**29**	None								Diffuse	
**30**	None								Negative	
**31**	**Exon 6**	c.632C>T	SUB	ACT > ATT	T > I	p.T211I	Yes	11	Focal	Substitution - Missense
**32**	**Exon 6**	c.639A>G	SUB	CGA > CGG	None	p.R213R	Yes	7	Diffuse	Substitution - coding silent
**33**	None								Diffuse	
**34**	**Exon 8**	c.867_868delCCInsTT	INDEL	CTC > CTT	None	p.L289L	Yes	1	Focal	Substitution - Missense
				CGC > TGC	R > L	p.R290L				
**35**	None								Diffuse	
**36**	None								Diffuse	
**37**	Exon 8	c.879G>A	SUB	GGG > GGA	None	p.G293G	Yes	4	Diffuse	Substitution - coding silent
**38**	**Exon 6**	c. 624C>T	SUB	GAC >GAT	None	p.D280D	No		Negative	
	**Exon 7**	c.692C>T	SUB	ACC > ATC	T > I	p.T231I	Yes	3		Substitution - Missense

Supplementary Table S8. TP53 mutation detection in patient samples with cSCCHN without neural involvement or cSCCHN with clinical PNI.
